# Enhancing Role Functioning in Clinical High Risk for Psychosis: An Open Trial of “InVEST” (Individualised Vocational and Educational Support and Training)

**DOI:** 10.1111/eip.70041

**Published:** 2025-04

**Authors:** Michelle L. West, Julia Pfluger, Shadi Sharif, Claire Goods, Michelle Friedman-Yakoobian

**Affiliations:** 1 Department of Psychiatry, University of Colorado School of Medicine, Aurora, Colorado, USA; 2 Beth Israel Deaconess Medical Center, Boston, Massachusetts, USA; 3 Department of Psychiatry, Harvard Medical School, Boston, Massachusetts, USA; 4 Department of Mental Health, Massachusetts Mental Health Center, Boston, Massachusetts, USA; 5 Brookline Center for Community Mental Health, Brookline, Massachusetts, USA

**Keywords:** clinical high risk, coaching, educational support, psychosis, role functioning

## Abstract

**Aim:**

School and vocational (“role”) functioning is an important treatment target for young people exhibiting signs of clinical high risk for psychosis (CHR-P). However, there are currently no evidence-based approaches to help people at CHR-P with role functioning difficulties.

**Method:**

This manuscript describes the development and pilot open trial evaluation of “InVEST” (Individualised Vocational and Educational Support and Training). InVEST is a coaching program designed to enhance role functioning in youth at CHR-P. InVEST is designed to be flexible, low-intensity, and easy-to-train, and it is carried out by bachelor’s and undergraduate-level “coaches” who work with clients individually for 4 months. The intervention specifically targets three mechanisms believed to relate to role functioning in this clinical population: (1) organisation, (2) task initiation, and (3) distress resilience. This manuscript describes a small open trial and the iterative development of a manual based on participant feedback about this intervention in preparation for a randomised controlled trial.

**Results:**

All participants (*N*=5) completed the baseline assessment, 16 weeks of coaching, and follow-up, with no dropouts. Participants reported high satisfaction with the program, and participant feedback was used to iterate and develop the InVEST manual. Additionally, most participants showed improvement in role functioning (4/5), organisation and planning (4/5), and task initiation (3/5) following InVEST participation.

**Conclusion:**

This small pilot study provides initial support for the feasibility and acceptability of InVEST and suggests that further study in comparison to a control condition is warranted.

Addressing role functioning impairments (difficulties with educational and/or vocational functioning) for youth experiencing early psychosis is an important treatment priority (Kern et al. 2008). Functional decline often begins early in psychosis (Niendam et al. 2009) and is evident in individuals at clinical high risk for psychosis (CHR-P; Cornblatt et al. 2007, 2012; Niendam, Bearden, Johnson, McKinley, et al. 2006). Youth experiencing CHR-P may present with mild changes in role functioning (i.e., difficulty focusing, decline in grades) to more notable declines (i.e., failing school courses, extended absences from school or work). Adolescents at CHR-P, even as early as age 12, have worse role functioning than their healthy adolescent counterparts (Carrion et al. 2013; Velthorst et al. 2018). Additionally, young people at CHR-P who develop a full psychotic disorder have worse role functioning than those who do not (Velthorst et al. 2018).

Interventions focused specifically on role functioning in CHR-P are needed. Functional decline may worsen treatment engagement and severity of early psychosis and other (e.g., depressive, anxiety) symptoms. Researchers have developed several evidence-based vocational interventions for individuals who have experienced first-episode psychosis (FEP), including individual placement and support (Killackey et al. 2008, 2006), supported education and employment (Mueser et al. 2015; Rosenheck et al. 2017), and cognitive adaptation training (Allott et al. 2017). However, these FEP interventions are not appropriately adapted to target role impairments in CHR-P from both a developmental and a symptom perspective. More specifically, clients at CHR-P are typically younger in age and at a less acute stage of illness. As a result, FEP role functioning interventions lack appropriate developmental adaptations. These interventions prioritise employment over academic success and involve intensive staff support (e.g., direct employer engagement), which may not align with CHR-P needs. A role-functioning intervention for CHR-P should incorporate developmentally appropriate language, recognise the continuum of human experience, and emphasise both academic and vocational skills relevant to young teens and emerging adults. Given the focus on a stepped-care approach in CHR-P (where intervention intensity increases based on need) developing a flexible, low-cost, and low-intensity intervention that can be delivered as a standalone service or integrated into coordinated speciality care will be essential (Hamilton et al. 2024).

Given the specific needs for support in role functioning for youth experiencing CHR-P, we developed a manual and conducted an initial pilot of “InVEST” (Individualised Vocational and Educational Support and Training), a novel, low-intensity, and low-cost intervention to improve role functioning for CHR-P. InVEST was developed as a four-month, individualised “coaching” intervention designed specifically for educational and vocational support in youth experiencing role functioning challenges related to CHR-P. InVEST builds upon prior research targeting role functioning impairments and real-world experience helping youth in a CHR-P specialised clinic (Friedman-Yakoobian et al. 2018). This intervention provides one-onon-one coaching support with a bachelor’s or undergraduate-level “coach” who works collaboratively with the client (and their clinical team when appropriate) to meet role functioning goals. InVEST focuses on three primary target areas believed to impact role functioning difficulties during the CHR-P stage: (1) organisation/planning (ability to effectively execute and organise work and manage distractions), (2) task initiation (ability to get started on tasks), and (3) distress resilience (ability to manage frustration or other challenging feelings while working to complete tasks).

We previously described the tenets of InVEST and its development at a community CHR-P clinic (West et al. 2021). Here we describe the first phase (open pilot trial, participant feedback, and manual development) of a treatment development study investigating the feasibility of InVEST. In this phase, we developed the InVEST manual via an iterative process, with refinements made based on real-world stakeholder feedback including feedback from participants as well as clinical and lived experience expert consultants (Onken et al. 2014).

## 1. Methods

### 1.1 Participants

Participants were young people (12-18 years old) who completed the Structured Interview for Psychosis-Risk Syndromes (SIPS; Miller et al. 2003) and met broad CHR-P criteria (Keshavan et al. 2011) plus at least mild role functioning impairment as measured by the Global Functioning Role scale (GF:R; Auther et al. 2006; Cornblatt et al. 2007).

### 1.2 Procedure

All study procedures were approved by the relevant Institutional Review Board. Before beginning the study, participants received an explanation of the study procedures and completed informed consent. Participants completed baseline assessments with a trained and reliable clinical interviewer. Clients were then assigned an InVEST “coach.” For this trial, coaches were bachelors’ level employees who were trained and supervised by the principal investigators (licensed psychologists) via an initial overview training workshop, access to a prior version of the InVEST manual, and weekly supervision. Clients met with their coach for 4 months via videoconference, phone, or in-person meetings, depending on client preference. Coaches worked flexibly with clients (either weekly for 1 h or multiple times per week for shorter periods) and worked on individualised InVEST goals, emphasising skills related to improving task initiation, organisation/planning, and distress resilience. After completing coaching, participants completed a four-month follow-up assessment and a participant feedback interview.

Throughout this open trial, the study team developed and revised the InVEST manual via an iterative process, incorporating feedback from participants and coaches. Additionally, two community expert consultants with clinical and lived experience expertise reviewed the manual, and their suggestions influenced the finalised InVEST manual.

### 1.3 Measures

#### 1.3.1 Psychosis-Risk Symptoms

##### Structured Interview for Psychosis-Risk. Syndromes (SIPS) and the Scale of Psychosis-Risk Syndromes (SOPS).

The SIPS/SOPS is a clinician-rated semi-structured interview and scale used to assess the intensity of CHR-P symptoms and diagnose CHR-P syndromes (McGlashan et al. 2010; Miller et al. 2003). This study involved administering the positive symptoms subscale. Each item is rated on a 0-6 scale, (0 =‘absent’, 3-5 = CHR-P range symptoms, and 6 = ‘severe and psychotic’).

#### 1.3.2 Role Functioning

##### *Global Functioning Role (GF:R) Scale and Global Functioning Social (GF:S) Scales* (Auther et al. 2006; Niendam, Bearden, Johnson, and Cannon 2006).

The GF:R/S are clinician-rated scales of role and social functioning developed specifically for CHR-P. The GF:R rates a person’s primary role (school, work). The GF:S assesses age-appropriate social relationships inside and outside the family. Both scales range from 1 (most severe impairment) to 10 (optimal functioning). Both scales have high interrater reliability (Cornblatt et al. 2007).

#### 1.3.3 Task Initiation

##### *Irrational Procrastination Scale* (IPS; Steel 2010).

The IPS is a 9-item self-report scale rated from 1 (“very seldom or not true of me”) to 5 (“very often true, or true of me”). It focuses on implemental attributes of procrastination with an emphasis on “irrational” delay (voluntary despite expecting negative consequences).

#### 1.3.4 Organisation/Planning

##### *Behaviour Rating Inventory of Executive Function-2 Self-Report and Parent Report* (Gioia et al. 2002).

The BRIEF2 for children (ages 5–18) is a self- and parent-report questionnaire of executive functioning in home and school environments. Its 86 items assess 8 constructs: inhibition, shifting, emotional control, initiation, working memory, planning/organisation, organisation of materials, and self-monitoring. It also contains two validity scales. The BRIEF2 is frequently used and has high internal consistency (Gioia et al. 2002). This study used Global Executive Composite (GEC) T-Scores, which incorporate clinical scales.

#### 1.3.5 Distress Resilience

##### *PROMIS* (Cella et al. 2010; Pilkonis et al. 2011; U.S. Department of Health and Human Services, National Institutes of Health 2010).

The PROMIS is a self-report questionnaire of anxiety, depression, and sleep disturbance on a five-point scale focused on the past week.

#### 1.3.6 Participant Satisfaction

##### *Client Satisfaction Questionnaire* (Attkisson and Greenfield 2004).

The CSQ is a direct, global measure of an individual’s experience with a service, with good internal reliability and construct validity. The CSQ was slightly modified to include 7 quantitative questions (e.g., satisfaction with quality of service, fit with needs, and overall satisfaction) and 3 qualitative questions (e.g., what was most helpful, suggested changes, additional comments).

##### Participant feedback interview.

The study team conducted participant feedback interviews to understand the participant experience with InVEST and obtain direct client feedback. Participants answered questions including: “What were some of the things/tasks you worked on with your coach?” and “Suggestions to improve InVEST?”

### 1.4 Analyses

Descriptive analyses investigated the demographic characteristics of the sample. Feasibility and acceptability were assessed by analysing the number of participants that moved from one stage of the study to another (screening to baseline, session attendance and engagement), the Client Satisfaction Questionnaire, and participant feedback interviews. Participant feedback interviews were qualitatively reviewed to investigate the specifics of participant experiences. Given the small sample for the open trial, target outcomes were analysed at a participant level by comparing baseline and follow-up data per participant and calculating percent change.

## 2. Results

### 2.1 Sample Demographic and Clinical Characteristics

The open trial included 5 participants, with a mean age of 15 (range 14-17). Three participants were white, one identified as mixed race, and one identified as Hispanic. Participants’ gender identity included cis-male (*n* = 1), cis-female *(n =* 1), agen- der *(n* = 1), preferred not to disclose *(n =* 1), and questioning or unsure *(n* = 1). All participants attended public school, ranging from eighth to eleventh grade. Participants’ self-reported goals included getting help with motivation, organisation, and focus for school and planning for college.

All participants exhibited role functioning difficulties with scores on the Global Functioning: Role (GF:R) scale ranging from 3 (marginal ability to function) to 7 (mild impairment). Scores on the IPS ranged from 22 to 38 (scores of 20 to 23 = bottom 10%, 24 to 31= bottom 25%, 32 to 36 = middle 50%, and 37+ = top 10%). Behaviour Rating Inventory of Executive Function-2 Self-Report (BRIEF 2) T-scores ranged from 59 to 90 (60 to 64 = mildly elevated, 65 to 69 = potentially clinically elevated, and 70+ = clinically elevated). Scores on the PROMIS 2.0 ranged from 58 to 85 (below 50 = within normal limits, 50 = average, 50 to 55 = mild, 60 to 70 = moderate, and 70+ = severe).

### 2.2 Feasibility, Acceptability, and Appropriateness

Six participants were screened for the InVEST open trial and five participants were eligible. All participants completed 4 months of coaching and all follow-up assessments. The number of sessions attended ranged from 8 to 18 sessions.

Most participants reported strong satisfaction with InVEST on the CSQ. All respondents reported acceptable levels of quality, meeting their needs, helpfulness, and satisfaction (see Table 1).

### 2.3 Qualitative Data: InVEST Participant Feedback Interview

The study team reviewed notes from participant interviews involving open-ended questions (see Table 2). There were several themes regarding what participants found to be most helpful, including: (1) support for improving organisation (5/5 participants), (2) coaches holding them accountable for work (5/5 participants), (3) motivation to complete work immediately (2/5 participants), and (4) support for communication (e.g., reaching out for help, disclosure) with teachers (2/5 participants). When asked “Is there anything you do regularly since participating in InVEST?”, four participants described still using organisation tools from coaching (e.g., Pomodoro timer to alternate work/breaks, calendars, etc.), and three participants responded that they felt more motivated to do work. In response to the question about suggestions for improving InVEST, one participant noted it may be beneficial for coaches to have active communication with teachers to better understand what may be happening in school, and another noted the importance of coaches adapting sessions to work on individual needs (both suggestions were incorporated into the manual).

### 2.4 Target Outcome: Role Functioning

**FIGURE 1 I:**
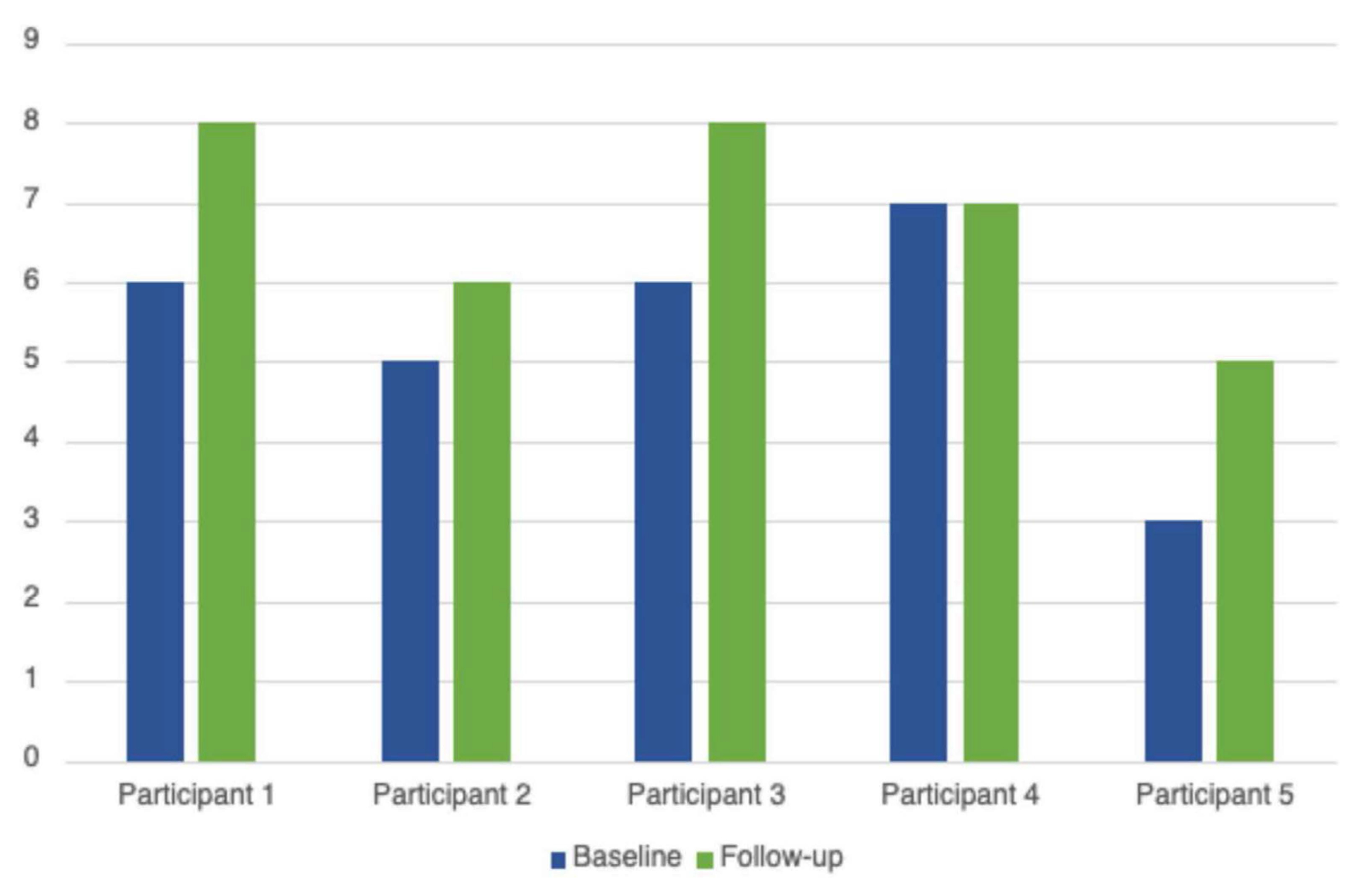
Role functioning ratings from baseline to follow-up across participants.

Between baseline and follow-up assessments, four of five participants exhibited improved clinician-rated role functioning scores (GF:R; Figure 1). One participant remained the same. Overall, there was an average of a 1.4-point increase across participants, which was a large effect size *(d* = −0.99).

### 2.5 Mechanisms: Organisation, Task Initiation, Distress Resilience

**FIGURE 2 I:**
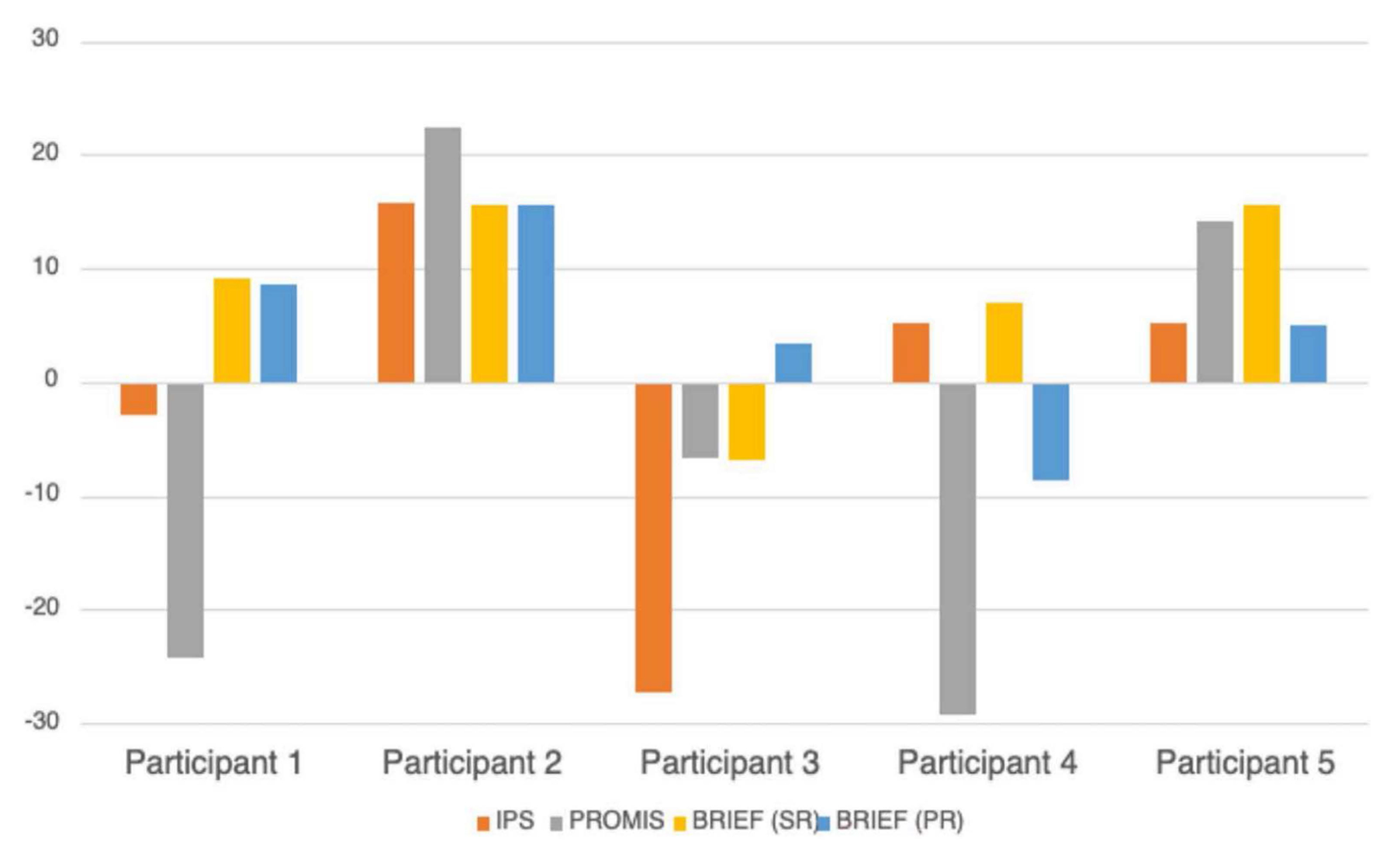
Percent change (baseline to follow-up) in mechanisms: organisation, task initiation, and distress resilience. Irrational Procrastination Scale (task initiation), PROMIS 2.0 (distress resilience), and BRIEF2 (Self and Parent Report; organisation/planning) scores were taken from followup and subtracted and divided by their baseline scores to calculate their respective percentage changes (the F-axis). Each per cent change was characterised as an improvement or worsening (according to scoring guidelines of the measure), with improvement represented as positive % change and worsening as a negative % change. All survey percentage changes are grouped by participant (labelled along the X-axis) to provide a holistic review of changes in target mechanisms.

Between baseline and follow-up assessments (Figure 2), most participants (4/5) exhibited improvements in organisation (BRIEF2, parent and participant report). There was an average 7.2-point decrease (improvement) in participant-reported BRIEF2 scores across participants, which was a medium effect size (*d* = 0.72). Similarly, parent-reported BRIEF2 scores exhibited an improvement for most (4/5) participants, with an average 3.4-point decrease across participants, which was a small effect size (*d* = 0.47). Most participants (3/5) also exhibited improvement in task initiation (IPS) scores with a small effect size (*d* = 0.11). Fewer participants (2/5) exhibited improvements in self-reported distress resilience (anxiety, depression, sleep disturbance; PROMIS) with a small effect size (*d* = −0.13).

### 2.6 Qualitative Academic and Vocational Outcomes

All five participants demonstrated diverse educational challenges and benefited from individualised coaching that targeted organisation, task initiation, and self-advocacy. Coaching sessions commonly addressed organisation through strategies such as assignment spreadsheets, to-do lists, and teacher outreach, which improved academic accountability and task management.

Coaches also explored participants’ vocational goals, with participants identifying and working toward future aspirations such as college preparation *(n* = 1) and career exploration (*n* = 1). Sessions provided structured support for goal setting, resume building, and skill development, such as note-taking and planning for long-term projects. Additionally, coaching fostered personal growth, with participants highlighting improvement in time management, motivation, and self-advocacy. Several participants reported that coaching increased their confidence in seeking support from teachers and parental figures (*n* = 4) and strengthened their ability to manage academic responsibilities (*n* = 4). Table 3 describes some examples.

Overall, these self-reported outcomes illustrate the InVEST program’s role in supporting both academic performance and vocational development, aligning with its targeted treatment mechanisms.

## 3. Discussion

This small feasibility and protocol development study provides initial support for justifying further study and development of InVEST as a specific role functioning intervention for youth experiencing clinical high risk for psychosis (CHR-P).

The initial review of the feasibility, acceptability, and appropriateness of InVEST was strong. All participants eligible for the study completed the baseline assessment, 16weeks of coaching, and follow-up assessments (zero dropouts). Additionally, all participants reported strong satisfaction with the program. They expressed appreciation for the tools provided to work on organisation and task initiation and stated that “most or all of their needs were met.” Overall, 4 out of 5 participants exhibited improved role functioning after InVEST, with an average 1.4-point increase across participants. When examining the specific treatment targets (organisation, task initiation, and distress resilience), there was evidence of improvement in mechanisms (with mixed results across variables). Overall, preliminary results suggest InVEST may engage two of three treatment targets (organisation and task initiation).

Quantitative and qualitative data gathered during this open trial were invaluable for the development of the InVEST manual in preparation for a randomised controlled trial of the intervention. Important changes to the InVEST manual informed by these data included adding cultural considerations to each section of the manual to provide coaches with a framework on how to approach clients from a culturally informed lens. Additionally, clinical vignettes were included in the skills sections to offer relatable and useful examples for coaches. Procedurally, another change following the open trial was increasing the eligibility age range to 22years (rather than 18), to serve a greater portion of the CHR-P population. This change allowed consideration for adapting InVEST for young adult clients and led to the inclusion of resources for college students and more content focused on supporting individuals entering the workforce. Feedback from coaches and consultants regarding the clarity and usefulness of the manual also led to further enhancements, improving usability. These critical changes drastically improved the InVEST manual and the training procedures for coaches.

This study has multiple limitations. This very small open trial did not include a comparison condition, so no determinations can be made on the impact of InVEST on role functioning or proposed mechanisms. All participants were engaged in some form of treatment (e.g., therapy, medication management), which may also impact role functioning. We cannot know whether role functioning improvements resulted from InVEST versus other treatments in this open trial. The extremely small sample size *(n* = 5) makes it impossible to draw any conclusive determinations from the data, and effect sizes are less stable with small sample sizes. Furthermore, all the participants in this study focused primarily on academic functioning, so further investigation of the impact of InVEST on vocational functioning is still needed. We are unable to generalise these findings to youth experiencing CHR-P in general, and the sample size is likely insufficient to detect significant effects of InVEST on treatment targets. Finally, there were some assessment limitations. Measurement of role functioning using the GF:R scale poses some challenges. The GF:R gives a single broad total score, which fails to separately capture aspects of role functioning (e.g., performance, support needed, time spent on role functioning tasks). The GF:R also adjusts scores for individuals receiving additional services (e.g., coaching), which creates complexity in rating appropriately. Overall, it is possible for participants to improve in aspects of role functioning while having the same GF:R score, resulting in difficulties capturing more subtle changes using this measure. There are also multiple possible interpretations of the more limited signs of improvement in distress resilience: the self-report measure used (PROMIS) may be insufficient for detecting changes in distress resilience, the small sample in this open trial may have contained few participants for whom distress resilience was a primary challenge, InVEST may need further refinement to target this skill or distress resilience may be better tackled in psychotherapy rather than in coaching.

Although this study has limitations, we are optimistic about these preliminary findings. The next steps for InVEST are to complete a randomised controlled trial with a larger sample size using the extended age range (up to 22). Through the randomised controlled trial, we aim to compare InVEST to a comparison condition (treatment as usual waiting list) to examine whether this intervention continues to appear to be feasible and acceptable and whether it appears to engage the treatment targets above and beyond treatment as usual. InVEST is not intended to be separate from or to replace CHR-P evidence-based treatments (e.g., Addington et al. 2005; Compton et al. 2007; Fusar-Poli et al. 2013), but to supplement treatment as an important component of CHR-P care (particularly as a “step one” less intensive treatment, or as part of CSC teams). With a larger sample, we will also have an opportunity to examine relationships between target mechanisms and overall role functioning improvement. We also intend to further refine the training process for InVEST coaches to expand access to this intervention. We will gather information about the characteristics of coaches to consider future implementation and generalisability of the intervention (e.g., whether coaches can be undergraduate interns, BA level research assistants, youth with lived experience, etc.). We will also further refine the training procedure for coaches and gather additional feedback from coaches about the usefulness of the updated InVEST manual for supporting both academic and vocational functioning of youth up to age 22.

## Figures and Tables

**TABLE 1 |:** InVEST Client Satisfaction Questionnaire.

Questions	Client responses
“How would you rate the quality of service you have received?”	• Excellent (*n* = 2) • Good (*n* = 3)
“To what extent has our program met your needs?”	• Almost all my needs have been met (*n* = 1) • Most of my needs have been met (*n* = 4)
“Have the skills and activities you received helped you deal more effectively with your difficulties?”	• Yes, they helped a great deal (*n* = 2) • Yes, they helped (*n* = 3)
“In an overall general sense, how satisfied are you with the services you have received?”	• Very satisfied (*n* = 2) • Mostly satisfied (*n* = 2) • Indifferent or mildly satisfied (*n* = 1)

**TABLE 2 |:** Participant feedback interviews.

Questions	Client responses
“What are some things that you worked on?”	• Organisation (*n* = 4) • Keeping up grades (*n* = 3) • Initiative and motivation for assignments (*n* = 2)
“What was the most useful part of InVEST for you?”	• Having someone to keep you accountable (*n* = 5) • Help with organising (*n* = 5) • Help communicating with teachers/others to get help (*n* = 2)
“Is there anything you do regularly since participating in InVEST?”	• Use organisation skills (*n* = 4) • Focus more on what makes me feel motivated (*n* = 3) • Procrastinate less (*n* = 2) • Communicate more with teachers (*n* = 2)
“What was the least useful part of InVEST for you?”	• I didn’t really need help on my school work (*n* = 1) • Some strategies did not work or were hard to use outside of sessions (*n* = 1) • Nothing, it was all decently beneficial (*n* = 2)
“Were there any ways your InVEST coach could have improved in terms of their work with you?”	• Make it more fun (*n* = 1) • Focus on motivation and how to build it consistently (*n* = 1) • Avoid getting off track during the session and help me stay more focused (*n* = 1)

**TABLE 3 |:** Descriptive participant outcomes.

Achievement	Description
Sports eligibility	Caught up on schoolwork to become eligible for playing sports and to avoid needing summer school
Teacher communication	Communicated with teachers about confusing assignments and began attending extra help sessions after school
Overcoming distress and avoidance of phone and video calls	Practiced videoconference and phone calls with their coach to prepare for an online college course
Understanding academic challenges	Identified past pitfalls leading to falling behind in schoolwork and planned strategies to stay on track
School accommodations	Collaborated with treatment team and parents to access better supports and accommodations through school

## Data Availability

Data from this study is available through the National Institute of Mental Health (NIMH) Data Archive (NDA), a secure data repository that facilitates data sharing and access to promote scientific research. Researchers can request access to the data by submitting an application to the NDA, adhering to the data use terms and conditions set forth by the NIMH. Once approved, access will be granted to de-identified datasets to support further research and analysis.
